# Trans-septal course of anomalous left main coronary artery originating from single right coronary ostium presenting with atrial fibrillation in a severely obese patient: a case report

**DOI:** 10.1186/s43044-020-00093-x

**Published:** 2020-09-21

**Authors:** Armando Ugo Cavallo, Emanuele Muscogiuri, Marco Forcina, Antonio Colombo, Flavio Fiore, Massimiliano Sperandio

**Affiliations:** 1 Division of Radiology, San Carlo di Nancy Hospital, GVM Care and Research, Rome, Italy; 2 Department of Biomedicine and Prevention, University of Rome “Tor Vergata”, Viale Oxford, 81, 00133 Rome, Italy; 3 Division of Radiology, University Hospital Sant’Andrea, University of Rome “La Sapienza”, Rome, Italy; 4 Division of Radiology, Policlinico Militare Celio, Rome, Italy; 5 Interventional Cardiology Unit, San Carlo di Nancy Hospital, GVM Care and Research, Rome, Italy; 6 Maria Cecilia Hospital, Cotignola, RA Italy; 7 Intensive Care Unit, San Carlo di Nancy Hospital, GVM Care and Research, Rome, Italy

**Keywords:** Left coronary artery, Trans-septal course, Anomalous origin

## Abstract

**Background:**

To present a case of anomalous origin of the left coronary artery evaluated with invasive coronary angiography (ICA) and ECG-gated coronary computed tomography (CCT).

**Case presentation:**

A patient (55 years old, male) with a past medical history of respiratory failure and atrial fibrillation underwent ICA to rule out coronary artery disease. Subsequently, the patient underwent ECG-gated CCT to evaluate a suspected anomalous aortic origin of the left coronary artery, since the interventional cardiologist was not able to properly identify the left coronary artery and its distal branches. CCT showed left coronary artery originating from the right coronary Valsalva sinus, coursing within the interventricular septum and emerging at the middle segment of the interventricular sulcus, where the left anterior descending and circumflex arteries originated.

**Conclusion:**

The case we presented highlights the value of ECG-gated CCT in the evaluation of coronary anomaly anatomy and thus risk stratification derived by proper coronary anatomy assessment. Although ICA was not helpful in the diagnosis, it also has a pivotal role regarding the therapeutic management of this condition.

## Background

Coronary artery anomalies are often incidentally detected during invasive coronary angiography (ICA) procedures (0.6–1.3%) [1]. ICA could be useful in the detection of the anomaly itself, but it also has several limitations regarding stenosis evaluation since specific experience and peculiar techniques are required even to catheterize the ectopic ostia. ECG-gated coronary computed tomography (CCT) is a technique that allows to precisely assess the origin of the anomalous vessel and the vessel course unambiguously, and it showed also a better diagnostic performance than ICA in coronary artery anomaly evaluation [2]. The case we present is emblematic regarding limits and clinical indications of the two techniques in a patient characterized by anomalous aortic origin of a coronary artery.

## Case presentation

A severely obese, 55-year-old male (BMI = 65.74 kg/m^2^) with a recent medical history of respiratory failure and atrial fibrillation (AF) was admitted to our institution for performing an ICA in order to assess the presence of coronary artery atherosclerosis underlying AF. Before the procedure, an echocardiogram was performed, showing no structural abnormalities; systemic arterial pressure was 140/78 mmHg, and heart rate was 88 with AF.

Written informed consent was obtained from the patient. Institutional Review Board was not needed in this case.

The interventional cardiologist was not able to evaluate the left coronary artery ostium, since no vessel was clearly opacified when contrast medium was injected in the left coronary sinus of Valsalva (Supplemental Material 1). The right coronary artery was evaluated properly and showed no significant atherosclerotic disease (Supplemental Material 2).

Thus, an ECG-gated CCT exam was performed with a 256-slice scanner (Revolution CT, General Electric, USA) to evaluate the left coronary ostium and, eventually, left coronary artery anatomy.

CT scan was performed after the intravenous administration of 50 ml of iodinated contrast material (Omnipaque 350 mg/dl, GE HEALTHCARE, USA) followed by a saline flush with AF. Images were acquired in 1 gantry rotation time (0.6 s) and post-processed with a noise reduction algorithm (Snap and Shot Freeze, General Electric, USA). Finally, images were analyzed on a dedicated workstation (Advantage Workstation 4.7, GE Healthcare, USA) using volume rendering (VR) and multiplanar reconstruction (MPR).

**Fig. 1:**
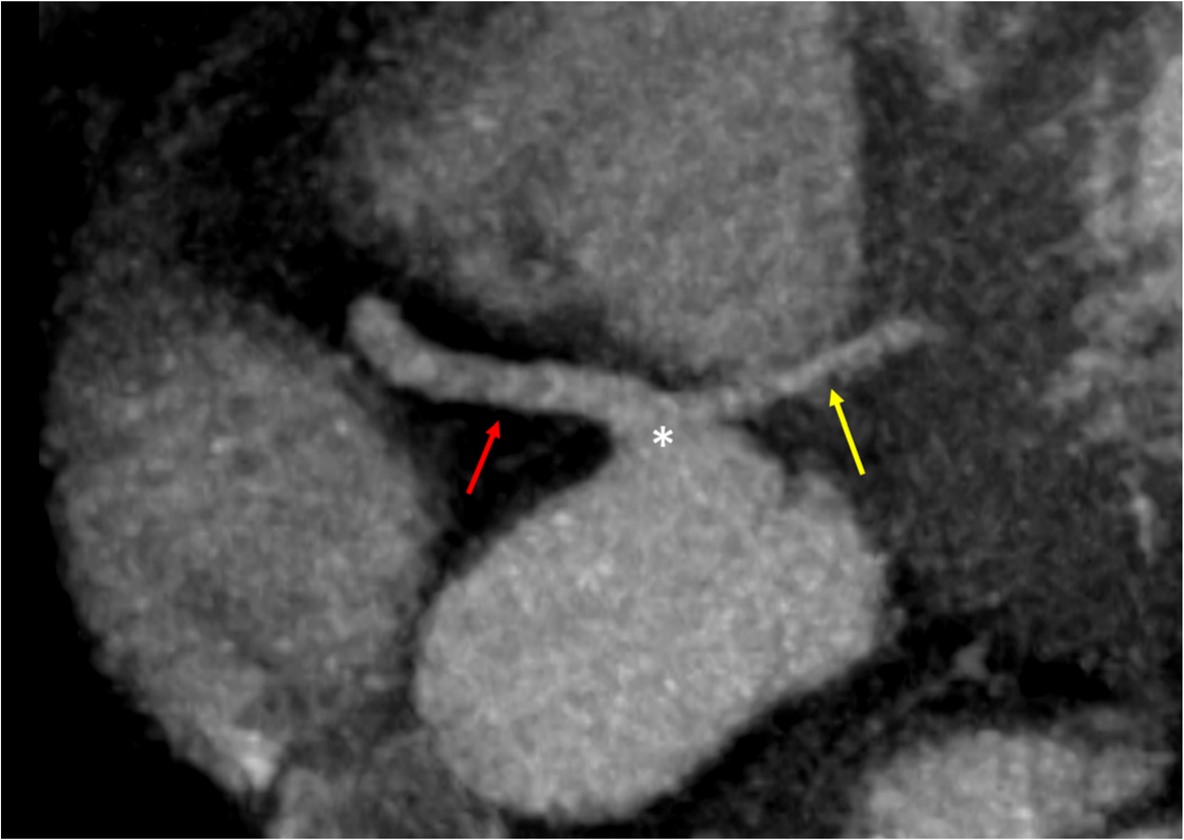
CCT MPR showing the right (red arrow) and left (yellow arrow) coronary arteries originating from the right coronary ostium (asterisk)

**Fig. 2:**
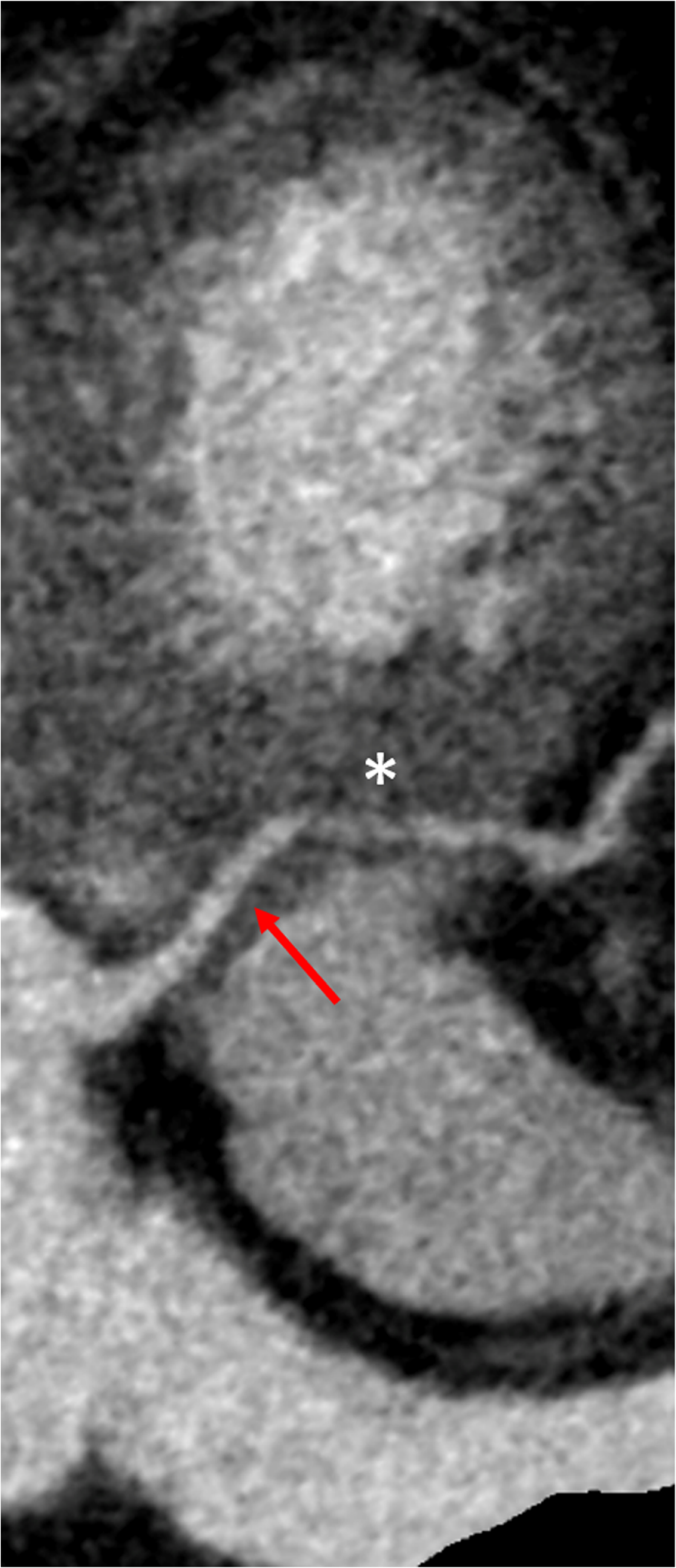
CCT MPR showing the left main coronary artery (red arrow) coursing in the interventricular septum (asterisk)

**Fig. 3:**
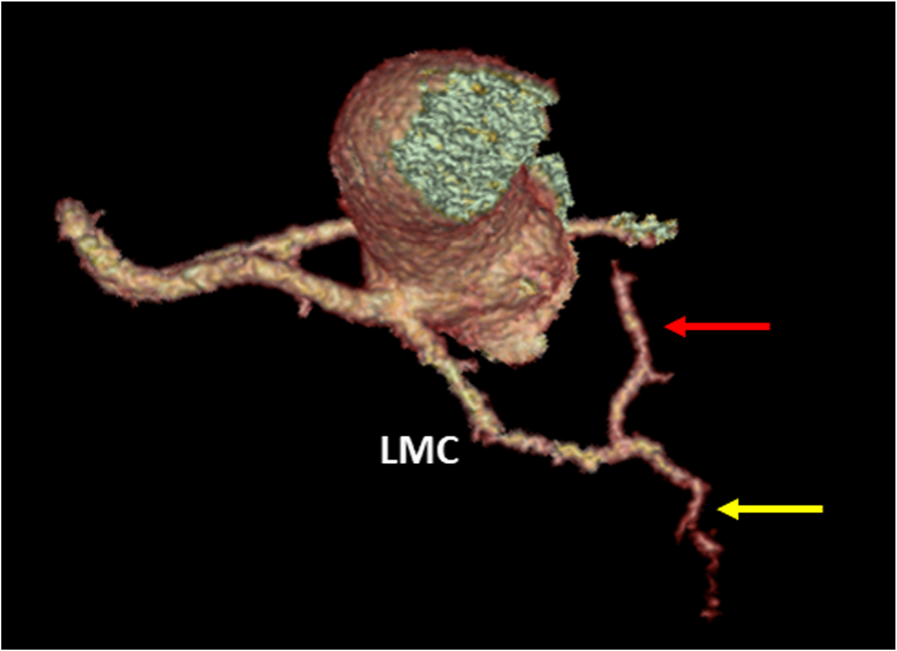
CCT VR reconstruction showing the left anterior descending artery (red arrow) and left circumflex artery (yellow arrow) originating from the left main coronary artery (LMC) after the intraseptal course

CCT showed the left coronary artery originating from the right coronary cusp (Fig. 1), with an intramyocardial course of about 2.4 cm within the interventricular septum (Fig. 2) with the left anterior descending artery (LAD) emerging at the middle segment of the anterior interventricular sulcus. The left circumflex artery (LCx) originated from the left main coronary artery distal segment (Fig. 3), coursing towards the atrioventricular sulcus, below the left atrial appendage.

Computed tomography confirmed an anomalous origin of the left coronary artery from the opposite sinus (L-ACAOS) with a trans-septal course.

The patient was treated with medical therapy and followed up after the ICA.

## Discussion

Coronary artery anomalies comprise several different congenital malformations and can be found in 0.3 to 5.6% of the population. They can be classified as benign and malignant depending on the vessel course and the potential risk of major cardiovascular events [3].

Trans-septal course of LAD is a malignant coronary anatomy variation since it is a potential cause of sudden cardiac death (SCD) in young athletes. Strenuous physical exercise in patients showing this anomaly has been, in fact, associated with SCD. Among the most important factors responsible for cardiovascular events, there is the dynamic compression of the artery during the cardiac cycle, leading to reduced blood flow during systole [4].

The clinical spectrum of L-ACAOS with a trans-septal course is wide and can mimic other acute heart conditions. Symptoms associated with this condition could be chest pain, syncope, dyspnea, and, as told before, SCD typically after physical exertion [4, 5].

The best non-invasive imaging technique to evaluate the anatomic features of L-ACAOS is ECG-gated CT angiography. This technique can show the most life-threatening features of the anomaly and has a high spatial resolution.

Including this case, there are 6 cases of the right single coronary artery with a subsequent trans-septal course of the left main coronary artery reported in the literature [6].

In the presented case, ICA was not able to show coronary artery anatomy, because the intraseptal tract of the LAD was probably associated to flow anomalies leading to reduced opacification after injection of contrast material in the right sinus of Valsalva. Thus, CCT played a major role in the assessment of coronary artery anatomy. The use of a 256-slice scanner with a very low rotation and acquisition time was of paramount importance for obtaining diagnostic images. In fact, AF often limits the diagnostic accuracy of CCT with conventional CT scanners.

Furthermore, to evaluate myocardial ischemia due to L-ACAOS, the best option is stress-ECG, to mimic physical exercise. Alternatively, an inotropic and chronotropic agent (e.g., dobutamine) with other non-invasive imaging techniques such as cardiac magnetic resonance (CMR) or echocardiography could be useful to simulate physical exertion [4].

Among invasive imaging techniques, intra-vascular ultrasounds (US) are the most useful for the evaluation of anatomical and physiological features of L-ACAOS is ICA, which is effective also in post-surgery follow-up [5].

The most effective and established therapy for this anomaly is surgery, with the un-roofing of the intramural segment and the creation of a neo-ostium, aiming to eliminate dynamic systolic compression, thus reducing the risk of myocardial ischemia and therefore SCD [4, 5].

## Conclusion

ECG-gated CCT performed with a 256-slice scanner with a very low rotation and acquisition time allowed to obtain good-quality images in a severely obese patient with AF and eventually to describe coronary anatomy.

## Supplementary information

**Additional file 1: Movie 1.** Left coronary ostium catheterization. After contrast material injection only a small, hypoplastic vessel is opacified.

**Additional file 2: Movie 2.** Right coronary ostium catheterization. Right coronary artery is well opacified and show no significant atherosclerotic disease.

## Data Availability

Not applicable

## Abbreviations

ICA: Invasive coronary angiography
CCT: Coronary computed tomography
AF: Atrial fibrillation
L-ACAOS: Anomalous origin of the left coronary artery from the opposite sinus
MPR: Multiplanar reconstruction
VR: Volume rendering
